# Effects of an Unexpected and Expected Event on Older Adults’ Autonomic Arousal and Eye Fixations During Autonomous Driving

**DOI:** 10.3389/fpsyg.2020.571961

**Published:** 2020-09-18

**Authors:** Alice C. Stephenson, Iveta Eimontaite, Praminda Caleb-Solly, Phillip L. Morgan, Tabasum Khatun, Joseph Davis, Chris Alford

**Affiliations:** ^1^Health and Applied Sciences, University of the West of England, Bristol, United Kingdom; ^2^Bristol Robotics Laboratory, University of the West of England, Bristol, United Kingdom; ^3^Human Factors Excellence (HuFEx) Research Group, and Centre for Artificial Intelligence, Robotics and Human-Machine Systems (IROHMS), School of Psychology, Cardiff University, Cardiff, United Kingdom

**Keywords:** autonomous vehicle, eye tracking, heart rate, human–machine interaction, human–machine interface, older adults, skin conductance level, trust

## Abstract

Driving cessation for some older adults can exacerbate physical, cognitive, and mental health challenges due to loss of independence and social isolation. Fully autonomous vehicles may offer an alternative transport solution, increasing social contact and encouraging independence. However, there are gaps in understanding the impact of older adults’ passive role on safe human–vehicle interaction, and on their well-being. 37 older adults (mean age ± SD = 68.35 ± 8.49 years) participated in an experiment where they experienced fully autonomous journeys consisting of a distinct stop (an unexpected event versus an expected event). The autonomous behavior of the vehicle was achieved using the Wizard of Oz approach. Subjective ratings of trust and reliability, and driver state monitoring including visual attention strategies (fixation duration and count) and physiological arousal (skin conductance and heart rate), were captured during the journeys. Results revealed that subjective trust and reliability ratings were high after journeys for both types of events. During an unexpected stop, overt visual attention was allocated toward the event, whereas during an expected stop, visual attention was directed toward the human–machine interface (HMI) and distributed across the central and peripheral driving environment. Elevated skin conductance level reflecting increased arousal persisted only after the unexpected event. These results suggest that safety-critical events occurring during passive fully automated driving may narrow visual attention and elevate arousal mechanisms. To improve in-vehicle user experience for older adults, a driver state monitoring system could examine such psychophysiological indices to evaluate functional state and well-being. This information could then be used to make informed decisions on vehicle behavior and offer reassurance during elevated arousal during unexpected events.

## Introduction

The private car is a vital element for the mental and physical well-being of older adults. Due to reduced mobility, the public transport system can be inconvenient and inaccessible (Broome et al., 2009). For example, challenges such as walking to a bus stop, or getting on and off a bus, can cause significant problems for adults with mobility issues. As such, driving provides access to local services, social events, and encourages participation in out-of-home activities. As well as the practical benefits to driving, research has indicated several affective advantages such as feelings of sensation, power, and youthfulness (Eisenhandler, 1990; Steg, 2005; Bergstad et al., 2011). However, age-related declines in cognitive, visual capacities, physical disability, and illness, subsequently impact driving ability as it becomes more physically and cognitively demanding. The possibility of becoming a non-driver rises with age (Anstey et al., 2006), and some drivers choose to restrict their driving (Dellinger et al., 2001). Driving cessation can have a negative impact on mobility and well-being, and feelings of isolation can be amplified (Qin et al., 2019). Some older adults find it more difficult to leave the home and stop participating in local or social activities (Marottoli et al., 2000), which in turn leads to a poorer quality of life. Consequently, ceasing driving can rapidly exacerbate physical, cognitive, and mental health challenges, and loss of independence.

Autonomous vehicles (AVs) promise to improve driving safety and efficiency by effectively removing the human from the driving task altogether. The role of the human is dependent on the level of autonomy of the vehicle. The Society of Automotive Engineering illustrated six levels of automation, ranging from 0 “No automation,” to 5 “Full automation” (SAE, 2018). While Levels 2 and 3 require a driver to monitor the environment and take back control of the vehicle when requested; Levels 4 and 5 requires little to no input from the driver. As different cognitive and physical demands of the task are replaced by automation elements, AVs may offer an alternative transport solution for the older population. By enabling a viable transportation option, mobility is likely to be restored enabling older adults to lead more independent lives (Smith and Anderson, 2017). In turn, this should promote participation in local and social events, encouraging feelings of social inclusion and satisfaction.

While the advent of AV technology offers many potential advantages for an aging population, the impact of the role as a passive driver on safe human–vehicle interaction and older adults’ well-being is not fully understood. Previous research has indicated the negative impact of partially automated vehicles on safe vehicle interaction, where the human is expected to stay ‘in-the-loop’ and take back control of the vehicle during expected or unexpected situations. Yet during Level 5 autonomous driving, the potentially negative consequences of a takeover request are eliminated due to the fully automatic capabilities of the vehicle. SAE (2018) refers to the in-vehicle user as a ‘passenger’ rather than a form of driver. Although the negative consequences of a takeover should be eliminated, previous research has demonstrated that full automation still has a significant impact on cognitive and affective functional state. From a cognitive perspective, studies have demonstrated that automation can increase mental underload and promote deficit attentional strategies (Young and Stanton, 2002). Automation has also been shown to encourage complacency and overtrust of a system (Parasuraman and Manzey, 2010), as well as increase frustration levels, particularly when automation cannot be overridden (Comte, 2000). These issues are potentially amplified in an older adult population with aging-related impairments, as they are more likely to rely on automated systems (McBride et al., 2011), find it more difficult to perform two or more tasks simultaneously (Kramer and Madden, 2008), and are more prone to lack understanding of advanced technology (Mann et al., 2007). Moreover, research has indicated that older adults have concerns using AVs due to issues related to trust and confidence, such as not having an operator nearby during failures (Faber and van Lierop, 2020). As such, some autonomous driving situations may initiate feelings of anxiety, and repeated activation of a stress response could be potentially damaging to their health and well-being (Cohen et al., 2007).

Considering the significant impact on a passenger’s functional state, a driver state monitoring (DSM) system including cognitive and affective indices to improve safety and well-being has been proposed (Collet and Musicant, 2019). A DSM system continuously monitors a user using a hybrid of measures including biological (e.g., muscle activity) and physical measures (e.g., blink frequency). By synthesizing and classifying functional state, the system can provide feedback to the passenger or adapt vehicle behavior. DSM systems have traditionally been applied during manual driving scenarios to detect fatigue and inattention. Situations such as night-time driving (Phipps-Nelson et al., 2011), prolonged driving (Finkleman, 1994), and extreme temperatures (Xianglong et al., 2018) can induce fatigue; whereas mobile phones (Strayer and Drews, 2007), in-vehicle systems (Arexis et al., 2017), and eating (Tay and Knowles, 2004) can induce inattention. In a manual driving scenario, a DSM system can use remote sensors to monitor fatigue behaviors such as prolonged eyelid closures and yawning. Upon detection of these behaviors, the system can warn the driver, or others, of the potentially dangerous situation.

Detecting fluctuations in cognitive and affective states with a DSM system has many potential benefits for improving passenger well-being and safety during Level 5 driving. The information about a passenger’s state could be used to modify in-vehicle information or vehicle behavior. For example, the in-vehicle system could provide reassurance at the appropriate time to reduce stress levels. It could also adapt vehicle behavior to improve comfort, e.g., leaving more headway between the vehicle in front. Alternatively, the system could identify the passenger’s cognitive load to present the optimum amount of feedback or information. For example, it could choose between auditory or/and visual feedback modalities depending on what the user is doing or how they are feeling. If the system detects lapses in attention, it could encourage automation monitoring. Manual driving research has provided promise toward the technical development of real-time unobtrusive sensors to detect driver state, however, additional studies are now needed to uncover the impact of *autonomous driving* scenarios on human cognition and arousal.

It is not possible to measure typical performance indicators of functional state during Level 5 autonomous driving as the passenger is not required to carry out manual driving behaviors (i.e., speed or lateral position changes). Capturing the human response in real-time may disentangle functional states during dynamic autonomous driving scenarios. To this end, most studies have utilized continuous measures such as eye gaze and indices of physiological arousal.

Cognitive underload and attentional deficits during automated driving have been demonstrated by measures of visual attention indexed by ocular behaviors. Visual strategy and the distribution of fixation points can provide information about where and when participants are shifting their attention. In general, eye gaze has been shown to be directed away from the driving environment (De Winter et al., 2014), and horizontal gaze dispersion is greater (Louw and Merat, 2017), when compared to manual driving, indicating lower situation awareness and reduced load. However, cognitive load and attentional allocation evolves over time with changing task demands. For example, Strauch et al. (2019) found that participants fixated in safety-critical areas (i.e., the steering wheel and forward roadway) more so during automated versus manual driving.

Several studies have attempted to understand the associations between constructs related to automation monitoring and attention itself. For example, participants with a high level of trust tended to monitor the road less (e.g., Helldin et al., 2013; Hergeth et al., 2016; Körber et al., 2018; Walker et al., 2019); and longer fixation duration and higher fixation count on the driving environment were associated with greater situation awareness (Shinohara et al., 2017). Considering the age-related differences in human-automation interaction, it is not clear whether similar relationships arise in older adult populations during Level 5 driving.

Suboptimal levels of cognitive functioning can also be assessed via psychophysiological measures of autonomic arousal (Lohani et al., 2019). Carsten et al. (2012) found that heart rate was lower during autonomous driving when compared to semi-automated and manual driving, providing further support for cognitive underload during periods of automation. Yet, manual driving is confounded by physical effort (e.g., moving the steering wheel, changing gears) and cardiac activity is likely to be modulated by motor demands (Laborde et al., 2017). Similar to studies measuring eye gaze, research into physiological indices have indicated that safety-critical events impact functional state. For example, Zheng et al. (2015) found that masseter electromyography increased, and self-reported comfort decreased, as the headway between the lead vehicle decreased. During unexpected takeover requests and misleading notifications, Ruscio et al. (2017) demonstrated an increase in sympathetic arousal measured by increased skin-conductance response amplitude. As increases in arousal have been linked to attention narrowing (e.g., Laumann et al., 2003), these results suggest that the breadth of attentional focus is limited during safety-critical events (Meinlschmidt et al., 2019). However, Ruscio et al. (2017) employed semi-automated driving with takeover requests. Therefore, participants were anticipating a takeover. This is distinct to Level 5 AVs where participants will not anticipate having any direct control of the vehicle.

Considering the potential AV benefits for older adults, such as maintaining mobility and independence, and the age-related individual differences related to human-automation collaboration, a comprehensive understanding of older adults’ psychophysiological state during periods of automated driving, particularly during safety-critical situations, is needed. Typically, research employs comparisons of autonomous driving to manual driving, but does not consider the distinct physical and cognitive demands. To this end, the aim of the present study was to investigate visual attention and autonomic arousal responses of older adults to a safety-critical event during a Wizard of Oz real-world autonomous journey. Participants experienced two types of stops: (i) one journey with the vehicle executing an unexpected stop due to detection of a ‘hazard’ (considered the safety-critical event) and, (ii) a different journey with an expected stop due to route set up in a repeated measures design with participants acting as their own controls. We monitored visual attention via fixation duration and fixation count, as well as physiological indices of electrodermal activity and heart rate. We also collected retrospective self-reported trust and reliability ratings in addition to summary qualitative feedback. We predicted the unexpected event would narrow the focus of overt visual attention coupled with an increase in autonomic arousal. This study formed part of the FLOURISH AV research project^1^ funded by Innovate UK, which studied older adults’ perceptions and interactions with AVs, including the development of an HMI, through co-design in a series of simulator and real-life studies.

## Materials and Methods

### Participants

Thirty-nine adults originally participated in this study. Two participants were excluded from all analyzes due to the AV experiencing technical errors during the journeys, leaving 37 participants (16 females, 21 males, mean age ± SD = 68.35 ± 8.49 years, range 48–89 years, two participants under 60 years). Due to recording errors during data collection, only 30 participants’ physiological data were subsequently analyzed (12 females, 18 males, mean age ± SD = 69 ± 8.75 years, range 48–89 years). Due to vision complications such as cataract (three), technical errors including unsuccessful calibration of the eye tracker (five), and low gaze samples (three), only 26 participants’ eye tracking data were subsequently analyzed (12 females, 16 males, mean age ± SD = 67.19 ± 7.32 years, range 52–89 years).

Five participants had corrected hearing. 17 participants were educated to degree level and 10 participants were working full- or part-time. All but three participants held a valid driving license, driving, and on average drove 2,500–4,900 miles a year. No participants had any previous experience with highly automated driving. Those with significant health conditions (e.g., epilepsy, neurological impairments, and coronary issues) were not permitted to take part. Participants received a £20 voucher as compensation for their participation to cover expenses. All participants gave written informed consent in accordance with the Declaration of Helsinki and were fully debriefed at the end of the study. Ethical approval was obtained by the Faculty of Health and Applied Sciences University of the West of England Research Ethics Committee (HAS.18.09.024).

### Apparatus

#### Autonomous Vehicle

**FIGURE 1:**
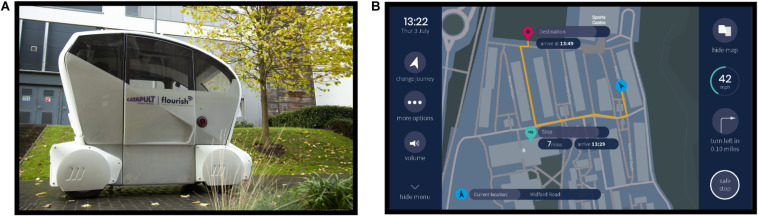
Autonomous vehicle and human–machine interface. **(A)** Autonomous Pod utilized during the study. **(B)** Human–machine interface display during the journey.

A Pod Zero autonomous pod provided by Aurrigo (RDM Group) was used as the AV (see Figure 1). The Pod is a compact research and development vehicle designed to be used in pedestrian areas and shared pedestrian/vehicle routes. It is electrically driven and can be used continuously for a period of 10+ hours of normal operation. It is a four-seater vehicle, with two benches facing each other designed similarly to a four-seater in a train. Due to safety regulations, a safety person was always present in the vehicle observing the environment and had access to an emergency stop button. Four marshals supervised the front and back of the vehicle, and the route was supervised by additional marshals at each intersection to ensure no vehicles or pedestrians caused an obstruction.

The autonomous behavior of the Pod was achieved using the Wizard of Oz approach (Kelley, 1985), whereby the Pod was remotely teleoperated in manual mode using a hand-held wireless control unit by an operator positioned behind the vehicle not in view of the participant. Driving the Pod in the teleoperated mode ensured that its actions were replicable between participants; the Pod could be made to respond similarly to different obstacles and followed the route as planned. At the beginning of the study, participants were told the vehicle was run fully autonomously. During debriefing, participants were told the Pod was operated manually by a teleoperator walking behind and remotely controlling it during the study. As the driving route involved a pedestrian area, the vehicle was controlled at walking speed, approximately 3–5 mph.

#### Human–Machine Interface (HMI)

The human–machine interface (HMI) was presented on a HannsG HT161HNB 15.6″ Multi Touch Screen connected to a Kodlix GN41 Mini PC (Windows 10, Intel Celeron processor, 8 GB RAM, 64 GB). The design of the HMI was informed by HMI design principles, public engagement workshops with older adults, and feedback from previous iterations of the HMI (Morgan et al., 2018; Eimontaite et al., 2019; Voinescu et al., 2020).

The HMI graphical touch screen displayed the vehicle speed, time remaining until destination, a safe stop button, a journey map, vehicle ‘health,’ and journey set up/change options (see Figure 1). The functionality of the safe stop button was described to the participant at the beginning of the study, emphasizing that pressing this icon would initiate the vehicle to stop. The vehicle ‘health’ icon provided information about the current working order of the automated system including the tires, brakes, network, and battery level. During the study, the vehicle health was always shown as being in good working order. The HMI presented visual and audio notifications to describe the vehicle’s behavior and journey course, such as “*Turning left*” and “*You have arrived at your destination.*”

### Journeys

Participants in the study went on six consecutive counterbalanced journeys. Before each journey, participants were provided with a scenario that specified the journey they were required to set up. There were six possible destinations/stops in total: Home, Health Center, Recycling Center, Sports Center, Sports Field, and Post Office. Among the six journeys there was always a journey including an expected stop, and another journey including an unexpected stop due to the ‘hazard.’ Both journeys were of an equivalent length and lasted for approximately 6 min. Some of the other journeys also included other variables such as picking up a friend. As the main focus of the current paper is to investigate the impact of an unexpected event, other journeys will not be described in detail. All journeys were randomized between participants so that the unexpected stop happened either during journey two or journey five, and the expected stop happened during journey one, journey three, or journey four. Overall, participants experienced approximately 60 min of the automated driving system.

The expected stop was initiated during journey set up and was therefore expected by the participants. A few seconds before the vehicle stopped, an HMI notification “*You are arriving at [Stop]*” was shown. Once the vehicle stopped, a notification “*You have arrived at [Stop]*” was shown. The HMI then displayed an option to either resume or stop the journey. All participants resumed the journey.

The unexpected stop was executed as an emergency stop appearing to the participants as happening suddenly, and as such, was not anticipated by the participants. A marshal was instructed to answer their mobile phone and walk in front of the Pod. The teleoperator of the Pod would then initiate the vehicle to stop. The HMI notification “*The vehicle detected a hazard in the road and has stopped. Your journey will resume shortly*” was presented on the touch screen. Once the marshal had moved safely out the way, the Pod would restart and continue the journey. The participant was not required to do anything.

### Procedure

**FIGURE 2:**
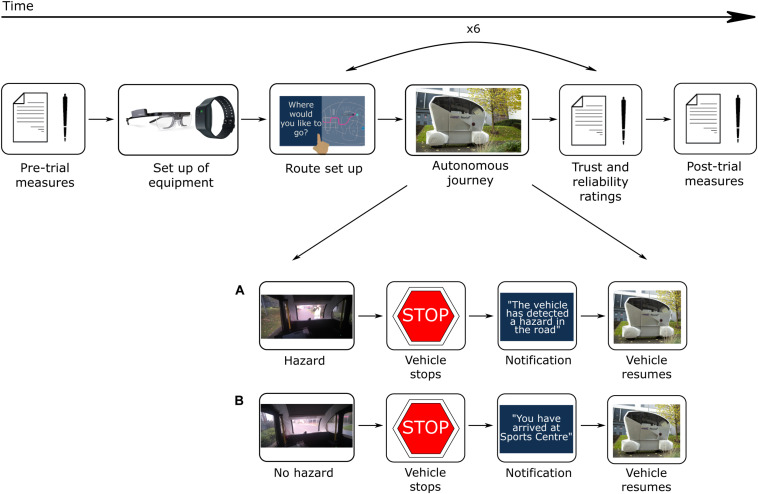
Experimental procedure. **(A)** During the unexpected journey event, a marshal walked in front of the vehicle. The vehicle stopped and displayed a message, *“The vehicle has detected a hazard in the road. The vehicle will resume shortly*.*”* The vehicle resumed once the roadway was clear. **(B)** During the expected journey event, the vehicle came to a stop when it reached a destination. A message displayed, such as, *“You have arrived at Sports Center.”* The participant was required to press *“Resume”* on the HMI for the vehicle to resume the journey.

Figure 2 shows a schematic of the experimental procedure. Participants arrived and met the researcher near the student accommodation area on the university campus, where the study took place. Participants were reminded of the content of the information sheet, asked about their well-being, and whether they had any concerns or questions. They were told that the study involved setting up a designated route on the HMI before experiencing AV journeys around the student accommodation area, for a total of six journeys. They signed printed copies of the consent form and filled in the paper pre-trial questionnaires. Next, they were shown images of the HMI and described the overall layout. Once the physiological and eye tracking equipment were set up, participants were taken outside and introduced to the Pod. Participants sat inside the vehicle wearing a seatbelt and facing forward. They were introduced to the safety driver but were advised not to converse with them. Likewise, the safety driver was told not to converse with the participant. Inside the Pod, participants were shown the HMI. At the beginning of each journey, the participant received the journey scenario that specified the journey destination and stop if there was one. Participants were required to set up the journey using the HMI. During the first journey, the researcher assisted them with setting up the journey and answering any questions they had. All participants successfully set up the journeys throughout the trial. Once the journey was set up, the vehicle started, and the journey began. Participants were told they could interact with the HMI as little or as much as they wished to. During the journey, the HMI would present notifications describing the journey process, such as *“Turning left.”* The HMI also displayed a navigation map showing the vehicle route (see Figure 1B). After each journey, participants provided verbal trust and reliability ratings to a researcher, including a reason for their rating. This process was repeated six times and all participants completed six journeys. Afterward, participants left the vehicle and filled in several post-trial questionnaires. The full testing session, including the induction and filling out questionnaires, lasted for approximately 150 min, depending on inter-individual variability. We found that a significant amount of time was required and needed to be scheduled when conducting studies with older participants. It was important to ensure a pace that did not increase fatigue, and enough time to reflect and discuss issues raised and answer questions.

### Measures

#### Trust and Reliability Ratings

We measured trust and reliability with a single-item scale to limit interruptions to the AV journeys. Participants were asked to rate how much they trusted the AV on a scale from 0 “Did not trust” to 10 “Completely trust.” They were also asked how reliable the vehicle was on a scale from 0 “Not reliable” to 10 “Completely reliable”. They were then asked to provide a reason for their rating. Ratings were taken verbally from participants at the end of every journey. The rating was also taken during the pre- and post-trial questionnaire phase, where participants were asked their current trust and reliability ratings of AVs on a paper questionnaire.

#### Physiological Signals

Continuous physiological acquisition of heart rate (beats per minute; BPM) and electrodermal activity (skin conductance level; μS) were collected using an Empatica E4 wristband (Empatica Inc., Cambridge, MA, United States and Milan, Italy) to measure levels of autonomic arousal. The sampling frequency for the electrodermal activity sensor was 4 Hz and the photoplethysmography sensor on the Empatica measured blood volume pulse at 64 Hz. The internal Empatica software derived the BPM. The Empatica E4 wristband was placed on participants’ non-dominant wrist to reduce the possibility of motion artifacts. The Empatica was fastened tightly as comfortable for the participant, so the wristband did not move around inducing artifacts. The E4 also collected acceleration data from a 3-axis accelerometer, which enabled monitoring of wrist movements. The sampling frequency of the accelerometer was 32 Hz.

An event marking button on the Empatica E4 was pressed in front of a camera, which triggered a LED light to be illuminated on the Empatica, and simultaneously logged a timestamp in the data. This mode of creating a marker was done to aid the later analysis of when events of interest (i.e., the unexpected stop) occurred in the physiological data.

#### Eye Tracking

Tobii Pro Glasses 2, an eye tracking device, was used to collect fixation metrics (Tobii Glasses Eye Tracker, Tobii Technology, Stockholm, Sweden). The Tobii Glasses are a wearable eye tracker worn like a pair of glasses. The design is lightweight and has no side or bottom frame, preventing any distraction in the participant’s visual field. The head unit is comprised of several cameras: a high-definition camera captured the participant’s field of view (82° horizontal and 52° vertical), and two eye tracking sensors below each eye captured participants’ pupil diameter and movements. To improve the accuracy of the eye tracking sensors, near-infrared lights illuminated the pupil. The sensors have a sampling rate of 100 Hz.

The Tobii Pro Glasses do not work with standard eyeglasses, as glasses can create additional glint that can lead to data corruption. Individuals wearing glasses were asked to remove them, and a suitable prescription lens from a set supplied as part of the Tobii kit was attached to the glasses. Once the participant was wearing the head unit, the manufacturer’s calibration procedure was followed which consisted of the participant fixating on a central target. This process typically took less than 30 s. In addition, participants were asked to view specific objects in the environment so that the accuracy and alignment of the system could be checked.

### Pre-processing

#### Physiological Arousal

Data were opened and pre-processed in Microsoft Excel 2016 using Excel’s in-built functions. Electrodermal activity and heart rate values, with corresponding timestamps, were pre-processed separately and followed the same procedure. For electrodermal activity (4 Hz sampling rate), every four samples were averaged to produce one value for every second, and similarly, 1 s averages were used to analyze heart rate data. The averaged data were aligned to the appropriate time point, to allow for averaging across time points of interest. Time points of interest were derived from timestamps in a video recording and the Empatica event marker. *Z*-scores were calculated to standardize the data due to the individual variability of physiological responses (Braithwaite et al., 2013) resulting in *z*-transformed skin conductance level (zSCL) and *z*-transformed heart rate (zHR). For data relating to the unexpected and expected stop, data were averaged within two times of interest: 30 s before the stop, and 30 s after the stop. 30 s was chosen as this is a standard epoch length used in vigilance and psychophysiological state monitoring research (e.g., Berry et al., 2015) and in other AV research investigating changes in physiology in response to events (Ruscio et al., 2017). This was also the minimum duration of recorded activity after the specific events that was not affected by other events such as the end of the journey.

#### Fixation Metrics

Eye tracking analysis was undertaken using Tobii Pro Lab software version 1.138 (Tobii Technology, Stockholm, Sweden). We first assessed the gaze sample percentage across the entire recording. The eye tracking glasses captured a mean of 80% (*SD* = 18%) of gaze samples.

Events were first logged to indicate the start and end of events in the recording. Times of Interests (TOIs) were defined by selecting the appropriate start and end event markers. This allowed for segmentation of the data into intervals of time relevant to subsequent data analysis. The ‘Pre-stop’ TOI was considered the 30 s before the presentation of the notification when the vehicle stopped; the ‘During’ TOI consisted of the time the notification was displayed visually (up to approximately 15 s); the ‘Post-stop’ TOI was considered the 30 s after the presentation of the notification. Gaze data from the recording were then manually mapped onto an image best depicting the overall visual view of the participant.

**FIGURE 3:**
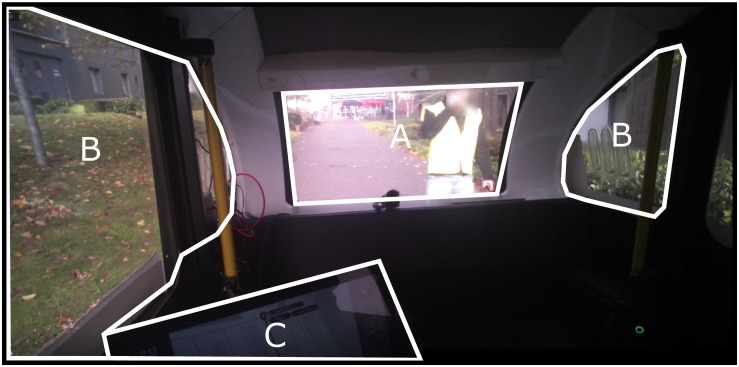
Areas of Interest (AOI) for eye tracking analysis. **(A)** Central environment. **(B)** Peripheral environment. **(C)** Human–machine interface.

Next, Areas of Interests (AOIs) were defined on each mapped image for each TOI (see Figure 3). Three AOIs were created representing the HMI, the central view of the driving environment, and the peripheral view of the driving environment. To finish, the ‘I-VT Filter (Fixation)’ was applied to the data, which set the velocity threshold parameter at 30 degrees/second. If the sample was below this threshold, it was classified as a fixation.

Total fixation duration and total fixation count metrics were exported from Tobii Pro Lab to Microsoft Excel 2016. Because the time of the critical event varied across participants, and to enable standardized comparison which took into account variability within patterns of fixations, it was necessary to calculate fixation count and fixation duration as a proportion of the total number of fixations and fixation durations. Fixation duration was defined as the amount of time spent looking at each AOI divided by the total duration of fixations. Fixation count was defined as the number of fixations toward each AOI divided by the total number of fixations. Averages were then calculated for subsequent analyzes.

## Results

All statistical analyzes were performed using IBM SPSS Statistics for Windows, version 26 (IBM Corp., Armonk, NY, United States). Descriptive statistics were performed, and normality was verified using the Shapiro–Wilk test and visualization of QQ plots of the unstandardized residuals. Assumptions of sphericity were tested using Mauchely’s test and, if violated, Greenhouse–Geisser estimates were used in the repeated measures calculations. The statistical threshold for significance was set to two-tailed *p* < 0.05. Effect size was reported as eta squared (η^2^) for one-way ANOVA significant results and partial eta squared ($\eta_{\text{p}}^{2}$) for two-way ANOVA significant results (Cohen, 1988). *Post hoc* analyzes were run with Bonferroni correction.

For trust and reliability ratings, a one-way repeated measures ANOVA (Journey: pre, unexpected, expected, and post) was undertaken. A 2 (Stop: unexpected and expected) × 2 (TOI: 30 s before and 30 s after) repeated measures ANOVA was performed to understand the impact of an expected and unexpected stop on heart rate and skin conductance level *z* scores. Two two-way repeated measures ANOVA were undertaken on both fixation count and fixation duration measures. The first was a 2 (Stop: unexpected and expected) × 3 (AOI: central, peripheral, and HMI) repeated measures ANOVA to understand the impact of journey type on AOI. The second ANOVA was a 2 (Stop: unexpected and expected) × 3 (TOI: pre-stop, during, and post-stop) repeated measures ANOVA to understand the impact of journey type on time.

### Trust and Reliability Ratings

The descriptive statistics are displayed in Table 1. For trust ratings, a significant repeated measures ANOVA [*F*_(2.__05_,_73.86__)_ = 15.05, *p* < 0.001, η^2^ = 0.295] with *post hoc* comparisons revealed that trust increased significantly from pre-all journeys to post-all journeys [*p* < 0.001], from pre-all journeys to the unexpected stop [*p* < 0.001], and from pre-all journeys to the expected stop [*p* < 0.001]. There was no significant difference in trust ratings between the unexpected and expected stop [*p* = 0.100].

**TABLE 1:** Mean (*SD*) of trust and reliability ratings over journeys.

**Subjective rating (0–10)**			**Journeys**	
	
	**Pre-**	**Post-**	**After an unexpected stop**	**After an expected stop**
Trust	7.11 (2.50)	9.22 (1.13)	9.16 (1.43)	9.00 (1.78)
Reliability	7.19 (2.39)	10.00 (0.88)	9.35 (1.18)	9.45 (1.02)

For reliability, the one-way repeated measures ANOVA model showed that the main effect for journey was significant [*F*_(1__.44_,_51.79__)_ = 25.56, *p* < 0.001, η^2^ = 0.415] and *post hoc* comparisons revealed that reliability ratings increased from pre-all journeys to post-all journeys [*p* < 0.001], from pre-all journeys to the unexpected stop [*p* < 0.001], and from pre-all journeys to the expected stop [*p* < 0.001]. Again, there was no significant difference in reliability ratings between the unexpected and expected stop [*p* = 0.100]. Overall, these findings indicate that subjective trust and reliability increased after AV experience and were not differentially impacted by the unexpected event.

### Heart Rate

**FIGURE 4:**
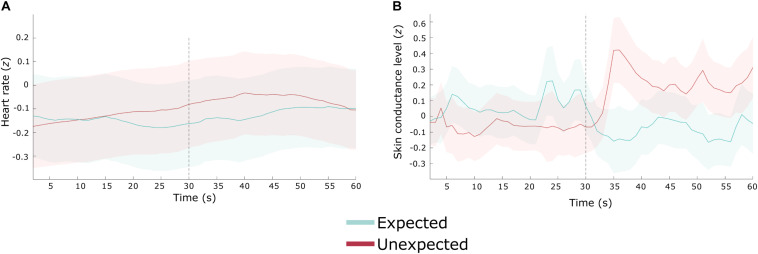
Heart rate and skin conductance level during an unexpected (red) and expected (blue) stop. Gray dashed line represents the time point the vehicle stopped. Shaded areas represent ± the standard error of the mean difference. **(A)** Heart rate (*z*-transformed). **(B)** Skin conductance level (*z*-transformed).

A 2 (stop: unexpected and expected) × 2 (time: pre-stop and post-stop) repeated measures ANOVA on zHR yielded no significant main effects of stop, time, or an interaction effect [*F*_(1,29)_ ≤ 0.56, *p* ≥ 0.461]. Heart rate was similar between the period before the expected stop [*M* = −0.15, *SD* = 1.01] and after the expected stop [*M* = −0.12, *SD* = 0.93]; and between the period before the unexpected stop [*M* = −0.13, *SD* = 0.95], and after the unexpected stop (*M* = −0.06, *SD* = 0.92). As illustrated in Figure 4, heart rate increased during the unexpected stop, but this did not reach statistical significance.

### Skin Conductance Level

The ANOVA model revealed no significant main effects for stop [*F*_(1,29)_ = 0.17, *p* = 0.684] or time [*F*_(1,29)_ = 0.37, *p* = 0.546]. However, the interaction effect was significant [*F*_(1,29)_ = 0.98, *p* = 0.019, $\eta_{\text{p}}^{2}$ = 0.176].

Pairwise comparisons revealed that during the unexpected stop, zSCL was greater following the stop [*M* = 0.16, *SD* = 0.83] when compared to zSCL preceding the stop [*M* = −0.05, *SD* = 0.72; *p* = 0.043]. There was no difference in zSCL before [*M* = 0.06, *SD* = 0.96] and after [*M* = −0.09, *SD* = 0.89] the expected stop.

In combination with the heart rate data, these results indicate that sympathetic arousal increased following vehicle cessation during the unexpected stop. See Figure 4 for a depiction of the skin conductance level response.

### Fixation Count

The 2 (stop: expected and unexpected) × 3 (AOI: central view, peripheral view, HMI) repeated measures ANOVA yielded a significant main effect of AOI [*F*_(1.42,35.55)_ = 27.74, *p* < 0.001, $\eta_{\text{p}}^{2}$ = 0.526], and a significant two-way interaction [*F*_(__1.52_,_38.1__0__)_ = 28.47, *p* < 0.001, $\eta_{\text{p}}^{2}$ = 0.532]. The main effect of the stop was not significant [*F*_(1,2__5__)_ = 0.44, *p* = 0.51; Table 2].

**TABLE 2:** Mean (*SD*) of fixation metrics count (%) and duration (%) across the human–machine interface (HMI), central environment, and peripheral environment during expected and unexpected stops.

**Fixation metric**	**HMI**		**Central environment**		**Peripheral environment**	
	**Expected**	**Unexpected**	**Expected**	**Unexpected**	**Expected**	**Unexpected**
Fixation count (%)	52.57 (30.07)	30.32 (28.23)	20.70 (20.02)	46.50 (26.35)	17.81 (20.85)	12.52 (14.76)
Fixation duration (%)	32.36 (27.75)	18.86 (23.06)	11.99 (13.86)	30.03 (22.55)	8.44 (11.54)	5.07 (5.97)

*Post hoc* comparisons of the two-way interaction revealed a higher number of fixations on the central environment during an unexpected stop compared to an expected stop [*p* < 0.001]; whereas fixation count was greater on the HMI area during the expected stop, compared to the unexpected stop [*p* < 0.001].

Overall, during the unexpected stop, the number of fixations were higher on the HMI area compared to the peripheral environment [*p* < 0.001], and the central environment compared to the peripheral environment [*p* < 0.001]. During the expected stop, the number of fixations were higher on the HMI compared to the central environment [*p* < 0.001], and the HMI compared to the peripheral environment [*p* < 0.001]. In combination, these results reveal that the number of fixations within the central environment was higher during an unexpected stop, whereas the number of fixations within the HMI was higher during an expected stop.

**FIGURE 5:**
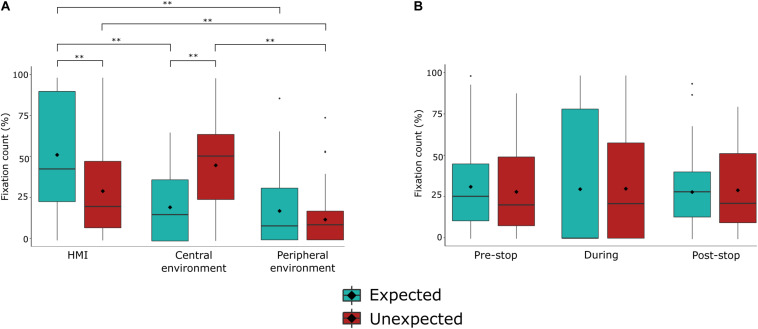
Fixation count (%) during expected and unexpected events (stops). **(A)** Fixation count during unexpected and expected journey events over areas of interest. **(B)** Fixation count during unexpected and expected journey events over times of interest. Bolded line represents the median value. Box represents the interquartile range. Vertical lines represent the lower/upper adjacent values. ♢ represents the mean value. **p < 0.001, *p < 0.05.

**FIGURE 6:**
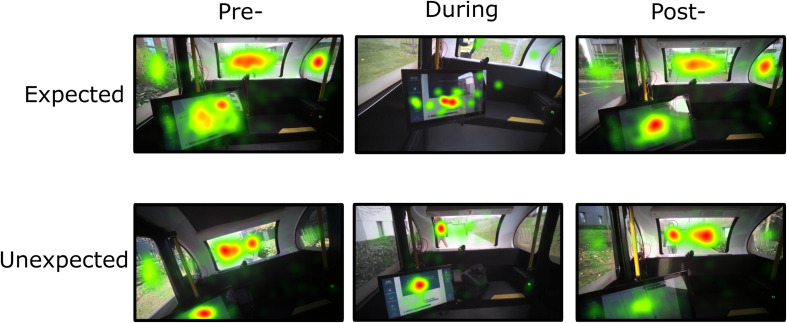
Total fixation count during expected and unexpected journey events (stops). The heat map represents the summary of all gaze points in the visual environment over three time points of interest. Colors indicate the total gaze fixations (fixation count increases from green – yellow – orange – red).

The 2 (stop: unexpected and expected) × 3 (time: pre-stop, during, and post-stop) repeated measures ANOVA revealed no significant main effects of time [*F*_(1.5__6_,_39.03__)_ = 0.88, *p* = 0.399] or stop [*F*_(1,25)_ = 0.44, *p* = 0.513], nor a significant interaction effect [*F*_(1.__21_,_30.21__)_ = 1.45, *p* = 0.244; Table 3]. See Figures 5, 6 for a depiction of the results.

**TABLE 3:** Mean (*SD*) of fixation metrics count (%) and duration (%) across pre-, during, and post- expected and unexpected autonomous journeys.

**Fixation metric**	**Pre-**		**During**		**Post-**	
	**Expected**	**Unexpected**	**Expected**	**Unexpected**	**Expected**	**Unexpected**
Fixation count (%)	31.94 (25.91)	28.87 (25.68)	30.17 (42.32)	30.43 (31.74)	28.97 (20.38)	30.04 (25.03)
Fixation duration (%)	17.36 (16.87)	17.58 (17.79)	19.34 (31.02)	19.51 (25.30)	16.08 (13.95)	16.88 (19.27)

### Fixation Duration

The 2 (stop) × 3 (AOI) ANOVA model yielded a significant main effect for AOI [*F*_(__1.59_,_39.72__)_ = 29.23, *p* < 0.001, $\eta_{\text{p}}^{2}$ = 0.539], and a significant interaction effect [*F*_(1.__58_,_39.37__)_ = 23.27, *p* < 0.001, $\eta_{\text{p}}^{2}$ = 0.482]. The main effect for stop was not significant [*F*_(1,2__5__)_ = 0.44, *p* = 0.516; Table 2].

*Post hoc* comparisons revealed that fixation duration on the HMI was longer during the expected stop compared to the unexpected stop [*p* = 0.003], but longer on the central environment during the unexpected stop compared to the expected stop [*p* < 0.001]. Fixation duration on the peripheral environment was marginally greater during the expected compared to the unexpected stop [*p* = 0.055]. Additionally, for the unexpected stop, fixation duration was shorter for the peripheral environment when compared to the HMI [*p* < 0.001] and the central environment [*p* < 0.001]. For the expected stop, fixation duration was longer on the HMI compared the peripheral environment [*p* < 0.001] and the central environment [*p* < 0.001].

The 2 (stop) × 3 (time) repeated measures ANOVA revealed a significant main effect of time [*F*_(__1.63_,_40.82__)_ = 6.52, *p* = 0.006, $\eta_{\text{p}}^{2}$ = 0.207]. The main effect for stop [*F*_(1,25)_ = 0.44, *p* = 0.516] and the interaction effect were not significant [*F*(2,50) = 0.21, *p* = 0.813]. Fixation duration was greater during the stop [*M* = 19.96, *SD* = 8.42] compared to after the stop [*M* = 16.48, *SD* = 7.72], regardless of whether it was an expected or unexpected stop [*p* = 0.01]. See Table 3 for an overview of the means and standard deviations.

**FIGURE 7:**
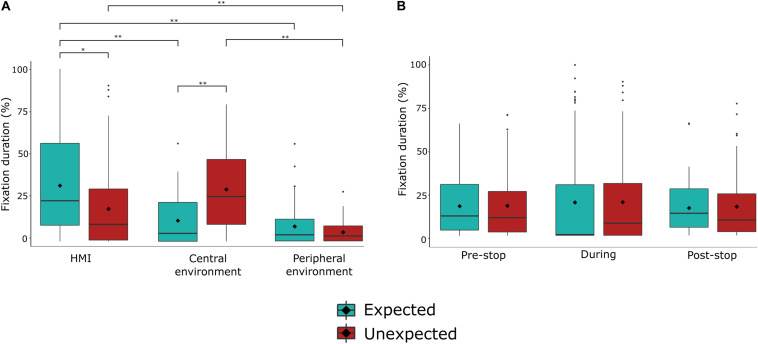
Fixation duration (%) during unexpected and expected journey events (stops). **(A)** Fixation count during unexpected and expected journey events over areas of interest. **(B)** Fixation count during unexpected and expected journey events over times of interest. ♢ represents the mean value. **p < 0.001, *p < 0.05.

**FIGURE 8:**
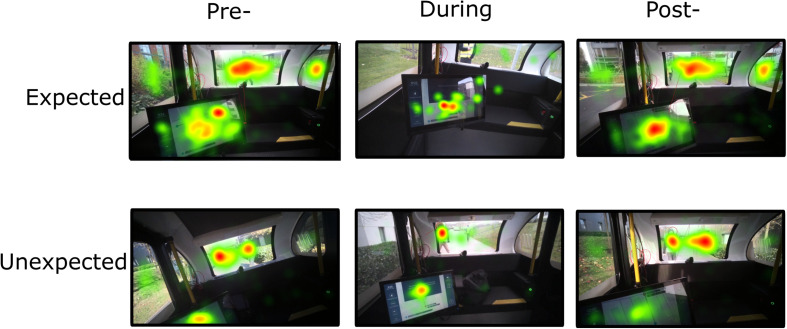
Total fixation duration during expected and unexpected journey events (stops). The heat map represents the summary of all fixations in the visual environment over three time points of interest. Colors indicate the total gaze fixation duration (fixation duration increases from green – yellow – orange – red).

In combination, these results suggest that while similar visual demands were afforded to the scene, participants visual attention was distinctly allocated during the unexpected and expected stops. Fixation duration was longer on the central environment during an unexpected stop, whereas fixation duration was longer on the HMI during an expected stop, indicating distinct visual attention resource allocation between the different types of stop. The results are depicted in Figures 7, 8.

## Discussion

This study sought to understand the impact of an unexpected event during Level 5 autonomous driving on gaze behavior, autonomic arousal, and associated trust levels. To accomplish this, an experiment was designed where the participants experienced what they thought were autonomous journeys that included two stops on separate journeys: one unexpected stop initiated by a ‘hazard,’ and one expected stop initiated as part of the planned journey set up. Elevated electrodermal activity persisted after the unexpected stop. Gaze fixation metrics revealed several visual behavior differences. Overall, participants searched the central environment, inclusive of the ‘hazard,’ for longer during the unexpected stop, whereas during the expected stop, the HMI area captured visual attention, as measured by greater fixation counts and longer fixation durations. Trust and reliability ratings also increased from pre-journey values and remained high after each type of journey.

The distribution and duration of fixations measured with eye tracking outline the differences between the two types of journeys: while in the unexpected stop journey participants had longer fixation durations and greater fixation count toward the central environment (containing the ‘hazard’), in the expected stop visual attention was directed toward the HMI. Visual behaviors between stops were similar over time, indicating similar demands on visual attention. General visual scanning behavior can be understood by fixation counts, as more shifts within a scene are associated with a greater frequency of fixations. Fixation duration can provide further insight by indicating visual attention demands. Basic visual processing research has demonstrated that fixation duration increases with visual scene complexity (Pomplun et al., 2013) and cognitive load (Rayner, 1998), and is linked to uncertainty (Brunyé and Gardony, 2017). As such, our patterns of results imply that during an unexpected stop, visual attention was directed toward the central environment containing the ‘hazard,’ with the participants searching for information, rather than focusing on other aspects of the scene. These results are similar to research studying ocular behavior during manual driving and hazardous situations. The variance of fixations decreased when presented with a critical situation (Chapman and Underwood, 1998). In contrast, fixation duration increased coming up to, and during, a critical situation (e.g., Chapman and Underwood, 1998; Underwood et al., 2005). In addition, a negative relationship between task demands during driving and visual scanning behavior has been demonstrated, i.e., higher task demands reduced the dispersion of visual scanning (e.g., Recarte and Nunes, 2003; Savage et al., 2013). Moreover, Guo et al. (2019) found that fixation frequency and duration increased during accident scenes reflecting increased anxiety.

Searching for information related to the unexpected event might be explained by increased anxiety (Guo et al., 2019): the narrowing of visual attention, focusing on the hazard, is a common feature of increased arousal and stress (Chajut and Algom, 2003; Gable and Harmon-Jones, 2010). Moreover, the physiological results show increased sympathetic arousal, following the unexpected stop. Skin conductance levels increased with vehicle braking due to the unexpected event. This increase persisted up to 30 s, yet there was no significant difference found during the expected stop journey. Driving studies have indicated that high skin conductance levels are modulated by various phenomena such as increased workload (e.g., Mehler et al., 2012), stress (e.g., Affanni et al., 2018), anxiety (Barnard and Chapman, 2018), and lower trust in automation (Morris et al., 2017; see Lohani et al., 2019 for a review). It is therefore difficult to infer specifically why skin conductance levels rose, other than reflecting an overall increase in sympathetic arousal. Trust ratings were high after all journeys, implying trust levels did not modulate sympathetic arousal. However, response bias, particularly following verbal ratings, may have led to an overestimation of self-reported trust. It should be noted that trust was measured retrospectively once the vehicle had successfully completed the journey. Factors such as trust, workload, and anxiety are time-varying, and as such, participants may have experienced lower trust levels during the journey, represented by heightened skin conductance. However, as the vehicle behaved appropriately to the unexpected event (e.g., braking and notifying the participant), and the journey completed successfully, this may have encouraged participants to rate their trust of the vehicle’s behavior positively at the end of the journey (Choi and Ji, 2015).

We did not find any statistically significant difference in heart rate although Figure 4 shows elevated heart rate, similar to skin conductance, for the unexpected stop compared to the expected stop. As skin conductance is regulated by the sympathetic nervous system, and heart rate is modulated by both the activation and suppression of sympathetic and parasympathetic branches of the autonomic nervous system, respectively (Thayer et al., 2010), our results suggest that the vehicle stopping in response to a unexpected event might reflect a mild sympathetic dominance. Ruscio et al. (2017) measured physiological responses to takeover requests following various warnings. During semi-autonomous driving, heart rate decreased relative to manual driving following reliable warnings, misleading warnings, and no warnings. Skin conductance response amplitude increased during misleading warnings and no warnings. They also found that respiratory sinus arrhythmia, an index of parasympathetic activity, increased from manual driving to an unexpected takeover with no warning. Their results reveal an imbalance between the parasympathetic and sympathetic branches during takeovers preceded by a misleading or no warning. The authors suggest that this discrepancy may reduce attentional capacity, resulting in cognitive overload. Although we did not measure specific or non-specific response amplitude changes, but rather changes in skin conductance level, our results are compatible as we found a similar moderate effect of increased skin conductance level following vehicle cessation without any warning (unexpected stop). However, our results are difficult to directly compare to Ruscio et al. (2017) findings as we did not measure physiological responses during manual driving or initiate a takeover request. We also did not separate parasympathetic activity from sympathetic activity; therefore, it is not clear whether a reduction in attentional capacity was associated with an increase in sympathetic activation as measured by an increase in skin conductance level.

A potential limitation in our study was the use of the Empatica E4 for assessing autonomic arousal. Gruden et al. (2019) recently found that manual driving-related movement artifacts impacted heart rate variability and skin conductance level measurements. Reasonable accuracy and reliability have been reported for this device providing wrist movements are low (Pietilä et al., 2017; Ragot et al., 2017), which was the case during our study, as Level 5 driving does not require behaviors such as changing gears. Nevertheless, we conducted additional analyzes and confirmed that accelerometer values did not differ between conditions (included in the Supplementary Material). In addition, conventional physiological research measures from the distal or intermediate phalanges of the ring and index fingers where there are a larger number of active eccrine sweat glands (Freedman et al., 1994; Boucsein, 2012). The E4, like many wearables, measures skin conductance via wrist sensors. As the wrist is less responsive to skin conductance, an underestimation of parameters is expected (van Dooren and Janssen, 2012; Payne et al., 2016). Despite this, the Empatica E4 was a relatively unobtrusive measurement device and was sensitive to changes in skin conductance level.

Despite the potential benefits of measuring sympathetic arousal and ocular behavior during Level 5 driving, it is not possible to avoid limitations inherent to skin conductance and eye tracking measurements. Due to a one- to four- second delay, or response latency, following a stimulus presentation (Boucsein, 2012), skin conductance measurements should not be used to detect time-critical events and are therefore not a usable metric on their own for a DSM system. In addition, it should be acknowledged that the skin conductance level values we measured were contaminated by skin conductance responses. If skin conductance responses were triggered by events during the journey, this would increase the underlying skin conductance level. Therefore, the values we report are impacted by both tonic and phasic responses to the events. Furthermore, it is well acknowledged that fixations cannot occur without attention, but attention can occur without fixations (Posner, 1980). Eye tracking is unable to detect the periphery of a participant’s visual gaze, but stimuli can be perceived pre-attentively in peripheral vision. Participants may have therefore discerned the notification and inhibited saccadic movement for further processing. Caution is therefore required in directly attributing changes in indirect measures, such as visual attention assessed with eye tracking, to direct measures of central attention. A robust DSM system may consequently benefit from including a variety of measures. The results obtained here do show significant differences in visual gaze behavior, perhaps reflecting changes in visual strategy as a result of reallocation of attentional resources relating to the unexpected event.

Although the current study attempted to produce increased ecological validity compared to laboratory studies, safety restrictions were put in place including the speed of the vehicle, the safety driver, and the marshals surrounding the vehicle. On average, the vehicle went between 3 and 5 mph. The speed of a vehicle has been shown to correlate with self-reported workload measures, i.e., the greater the speed, the greater self-reported workload (Fuller, 2005). However, research has found that this depends on the situation complexity. Low-complexity environments including motorways at faster speeds, or high-complexity situations including town centers at lower speeds, may modulate load in a similar manner (Paxion et al., 2014). In our study, the vehicle drove around a pedestrianized area, where the speed limit was 10mph. The vehicle shared the lane with pedestrians, cyclists, and obstacles such as bollards. Therefore, driving at a greater speed would not have been possible nor realistic or safe, even during manual driving.

Finally, the results imply that the unexpected event placed significant demands on attentional resources. However, eye tracking is an indirect measure of attention, and as the study mimicked Level 5 autonomous journeys, no direct performance measure could be derived to support this view. Yet, all participants were introduced to a “safe stop” button on the HMI, which could be pressed at any time if they wanted the vehicle to stop. None of the participants activated the safe stop. They could also have accessed the “vehicle health” icon, providing information about the overall health of the vehicle. None of the participants accessed this icon during the times of interest. Taken together, these findings suggest that the unexpected stop was not perceived as particularly dangerous as neither subjective reports nor subjective ratings, or all physiological indices reflected an extreme response that may be associated with more imminent or extreme danger. This is supported by summary qualitative analysis where 12 of the participants, representing around a third of the sample, expressed unease (e.g., “nervous it would not stop, would like a horn”) when discussing the unexpected stop with the researchers after journey completion. A further 3 expressed ease or confidence (e.g., “had stopped before, would this time”) in relation to the event, with the remainder simply noting that the vehicle had spotted the ‘hazard’ and stopped, performing its intended functions. In addition, as our study only included one unexpected event, further investigations using different types of unexpected events are needed to be able to characterize functional states to specific safety-critical scenarios.

Although our results are supported by the above-mentioned driving studies, the study presented in this paper varies considerably as our participants were not active manual drivers in control of the vehicle. As ocular behavior and motor execution are intrinsically linked both spatially and temporally, active drivers successfully fixate directly at the objects being interacted with or ones that precede the action. Despite these differences, our results are in agreement with Strauch et al. (2019) who investigated eye gaze of passengers during real-world autonomous driving. They found a greater frequency of fixations on safety-relevant AOIs when joining a highway during an autonomous journey when compared to manual driving and to the rest of the route. Visual scanning behavior was therefore affected by safety-critical situations regardless of active involvement in the driving task. Strauch et al. (2019) participants’ mean age was 23 years, and so our results extend this earlier research and suggest that older adults display broadly similar ocular behaviors to younger adults during safety-critical situations. However, our study does differ, as passengers could interact with an HMI throughout the journeys. Unlike a passenger in a manually driven car, in a Level 5 vehicle the monitoring of the automated system can take more significance, given the reduced need to pay attention to the road ahead. Despite this, we found that attentional focus narrowed toward the ‘hazard’ before, during, and after the critical event, which was also accompanied by an increase in skin conductance reflecting increased sympathetic nervous system arousal following the vehicle response.

Repeated stress response activation and consequential negative emotions may have a significant impact on overall health and well-being. Therefore, a DSM system could react in response to detecting increased arousal by either modifying the vehicle’s behavior, alerting the driver, or modifying HMI notifications and providing status updates or information about future events. For example, the HMI might adapt safety-related notifications to make them more engaging, multimodal, and alerting, depending on individual characteristics and the attentional level of the passenger (ranging from inattentive to over-alert). Moreover, the vehicle could learn the types of situations that have a negative impact on passenger well-being and adapt vehicle route or driving style to avoid them.

Taken together, these results have several critical implications for the safe implementation of Level 5 AVs for older adults. Our results reveal possible narrowing of visual attention and heightened arousal during an unexpected event as demonstrated by increased sympathetic arousal and a smaller distribution of fixations, coupled with an increase in fixations toward the unexpected event. In combination with consistently high trust ratings, these results suggest that the passive process of automated driving may restrict the focus of visual attention and heighten adverse responses. This study also demonstrates that the physiological indices examined can be useful and practical measures for evaluating passengers’ functional state during real-world autonomous driving. As such, a DSM system that includes these measures might be able to detect these behaviors and make an informed decision on vehicle behavior and adapt HMI notifications accordingly. The potential for negative experiences during Level 5 driving, coupled with human limitations in sustained monitoring during low and high arousal situations, suggests that a DSM system may be a necessary adjunct to fully AVs in supporting potentially vulnerable people in unexpected situations.

## Data Availability Statement

The raw data supporting the conclusions of this article will be made available by the authors, without undue reservation.

## Ethics Statement

The study was reviewed and approved by the Faculty of Health and Applied Sciences University of the West of England Research Ethics Committee. The participants provided their written informed consent to participate in this study.

## Author Contributions

AS, IE, JD, and TK collected and processed the experimental data. IE was responsible for running the study. CA, PC-S, and PM were involved in the planning and design of the experiments. CA and PC-S were involved in the implementation and supervision of the work. AS performed the analysis, drafted the manuscript, and designed the figures. CA, IE, and PC-S aided in interpreting the results and edited the manuscript. All authors commented on the manuscript.

## Conflict of Interest

The authors declare that the research was conducted in the absence of any commercial or financial relationships that could be construed as a potential conflict of interest.

## Footnotes

1 http://www.flourishmobility.com
