# Supplementary material for: Effects of an Unexpected and Expected Event on Older Adults’ Autonomic Arousal and Eye Fixations During Autonomous Driving
Source: Front Psychol. 2020 Sep 18;11:571961. doi: 10.3389/fpsyg.2020.571961 (PMC7531228; doi:10.3389/fpsyg.2020.571961)
Supplement: Supplementary file 1 [file Data_Sheet_1.pdf]

Supplementary Material

1 Movement index

Movement index was calculated to determine whether there was a difference in wrist movement between the expected and unexpected stop. The times of interest were the same as the physiological analyzes: 30 seconds before the stop and 30 seconds after the stop. Movement index was calculated by deriving the Euclidean distance between the current and previous x, y, and z coordinates. To compare the movement between stops, a 2 (stop: unexpected, expected) x 2 (time: pre-stop, post-stop) repeated measures ANOVA was conducted. The results revealed no significant differences between the main effects of stop and time, and the interaction effect ($F_{(1, 29)} \leq 2.10$, $p \geq .159$). Movement was similar before the expected stop ($M = 0.03$, $SD = 0.01$), after the expected stop ($M = 0.02$, $SD = 0.02$), before the unexpected stop ($M = 0.03$, $SD = 0.01$), and after the unexpected stop ($M = 0.03$, $SD = 0.01$).
